# Progress in Surgery of the Intestines

**Published:** 1903-09-19

**Authors:** 


### Progress in Surgery of the Intestines.

**Enteroptosis**, or "falling of the viscera," is considered by Lea,[1] who gives observations on 56 cases. The condition in some form is found in nearly 10 per cent. of women examined. The principal etiological elements in the production of the displacement of the viscera appear to be the following:—1. Impairment of the general health and feeble muscular development. 2. Increased size of the abdominal cavity, as in pregnancy, tumours, etc. 3. The influence of corsets—tight lacing. 4. Influence of peritonitis. Adhesions form, interfering with peristalsis, and the abdomen rarely recovers its normal muscular tone and elasticity. 5. Certain peculiarities in the shape of the thorax, especially a long narrow chest, with a very acute costal angle. Extreme enteroptosis may exist without calling forth any special symptoms, and there is no definite relationship between the amount of displacement of the viscera and the severity of the symptoms. The symptoms may be grouped under four headings:—1. Asthenic phenomena—the result of weakness and malnutrition. 2. Meso-gastric phenomena, resulting from traction on the mesentery. Sensations of weight and dragging, much aggravated by standing or any form of exertion. 3. Gastro-intestinal phenomena—constipation, colitis, and chronic dyspepsia the result of gastro-duodenal dilatation with vomiting, flatulence, and loss of appetite. 4. Nervous phenomena of a neurasthenic type. As regards diagnosis, it will be noticed that if the patient stands erect the lower part of the abdomen bulges forward, overhanging the pubes, while there is marked epigastric flattening. The patient is usually thin, so that the various organs can be palpated and their degree of mobility or displacement noted; but in some cases after the menopause there may be a considerable deposit of subcutaneous fat. The main object of treatment must be to increase the muscular tone and elasticity of the abdominal wall. This is best accomplished by the persistent use of gymnastic exercises after the Swedish method. If this is not possible an endeavour must be made to provide some artificial means by which effectual support to the viscera may be given and the downward displacement prevented. As the condition is often associated with debility and malnutrition, general tonic treatment is required. Operative procedures for the cure of enteroptosis are only justifiable if careful trial of other measures has failed to give relief. Many cases of so-called "movable kidney" are in reality examples of general enteroptosis, and nephropexy has often been carried out unnecessarily and without effecting a cure of the symptoms. In cases of hepatoptosis the liver has been sutured to the abdominal wall after making a raw surface on its convex border. If there is evidence of cholelithiasis or biliary cirrhosis, the ordinary operation of cholecystotomy is sufficient to produce fixation of liver and gall-bladder. In cases of intractable gastroptosis the stomach has been fixed to the abdominal wall, or the gastro-hepatic and gastro-phrenic omenta have been shortened. If the gastro-duodenal dilatation is extreme, the operation of gastro-enterostomy has been carried out successfully. Keith,[2] in a series of able lectures, discusses the nature and anatomy of enteroptosis. When the respiratory balance is upset the viscera, not only of the abdomen, but also of the thorax, are displaced downwards. Thus enteroptosis is the result of a vitiated method of respiration and should be assigned really to the respiratory diseases. It sometimes happens that the incidence of the disease falls particularly on one organ, very frequently the kidney, sometimes the stomach, liver or spleen. In such cases, although one organ has suffered the chief attack of the inspiratory force, yet a full examination will show that all are displaced although to a less degree.

**Intestinal Obstruction.**—Bowlby[3] deals with cases which simulate this condition, inasmuch as they present the same cardinal symptoms of pain, vomiting, and obstruction to the passage of wind and fæces. Amongst these conditions he mentions peritonitis, affections of the solid viscera as in renal and gall-stone colic, acute pancreatitis, embolism of the superior mesenteric artery, functional constipation and enteritis. He suggests the following points as to procedure when in a doubtful case it is decided to open the abdomen in order to seek for a supposed mechanical obstruction :—1. Open the abdomen in the middle line below the umbilicus, because most of the causes of obstruction will be found in the lower half of the abdomen. 2. Without allowing any intestine to escape examine first with two or more fingers, or even the whole hand, the right iliac region, and pass from there towards the umbilicus to feel whether there are any adhesions there. It is in this right lower half of the abdomen that most of the causes of obstruction are to be found, for here are (1) the appendix; (2) intestinal diverticula, perhaps attached to the umbilicus or to the neighbouring mesentery; (3) the commonest site for volvulus, that is the cæcum; (4) the usual site for the lodgment of an impacted gall-stone, that is, the lower part of the ileum; (5) a common place for adhesions due to caseous mesenteric glands; (6) the sites of inguinal, femoral, and obturator hernia. Further, if the obstruction be in the small intestine it is in the right iliac fossa that undistended intestine will be found, and if this can be secured and traced upwards it is the surest guide to the seat of obstruction. 3. Examine next the left iliac region and the pelvic region, the latter especially if the patient be a woman, for there, as additional causes of adhesions, may be inflamed ovaries or tubes or some uterine trouble with neighbouring inflammation. 4. If no cause can be discovered, then either open a coil of distended intestine and suture it to the skin, if the patient be too ill to bear more; or else decide to take the distended bowel out of the abdomen altogether, and if necessary open it, empty it, and suture it. It is only by so doing that you will be able to return it once you have decided to let it escape, and it is often only by so doing that you will find a deeply-seated obstruction. Interesting cases are reported by Pollard[4] and Taylor.[5]

**Duodenal Ulcer.**—Murphy and Luff[6] say this affection occurs most frequently in the fourth decade, and the great majority occurs in males. Many theories have been advanced as to their causation, but the most widely-accepted one ascribes them to the same cause as gastric ulcer—the effect of the gastric juice upon a circumscribed portion of the mucosa, the vitality of which has been impaired. In many cases there is no evidence of duodenal disease prior to perforation. Operation should be undertaken at the earliest possible moment after the onset of the acute symptoms, and must, in the majority of cases, be at first of an exploratory character; if duodenal perforation be suspected the incision should be through the right rectus muscle. Suture is preferable to excision of the ulcer, and can easily be performed as the first portion of the duodenum, in which the great majority of perforating ulcers are situated, is readily accessible. A successful case of operation for this condition is recorded by Burgess.[7]

[1] Med. Chron., July, 1902, p. 225. [2] Lancet, March 7, 1903, p. 633. [3] Brit. Med. Jour., Jan. 3, 1903, p. 5. [4] Lancet, March 28, 1903, p. 871. [5] Dublin Med. and Surg. Jour., March, 1903, p. 164. [6] Med. Chron., Nov., 1902, p. 134. [7] Lancet, April 18, 1903, p. 1098.

*(To be continued.)*

